# Differential Nanoscale Topography Dedicates Osteocyte-Manipulated Osteogenesis via Regulation of the TGF-β Signaling Pathway

**DOI:** 10.3390/ijms23084212

**Published:** 2022-04-11

**Authors:** Jingyuan Cui, Yaru Yang, Peiru Chen, Ruiqiang Hang, Yin Xiao, Xueting Liu, Lixin Zhang, Hui Sun, Long Bai

**Affiliations:** 1 Key Laboratory for Ultrafine Materials of Ministry of Education, State Key Laboratory of Bioreactor Engineering, East China University of Science and Technology, Shanghai 200237, China; cjy@ecust.edu.cn (J.C.); liuxueting@ecust.edu.cn (X.L.); lxzhang@ecust.edu.cn (L.Z.); 2 Frontiers Science Center for Materiobiology and Dynamic Chemistry, College of Materials Science and Engineering, East China University of Science and Technology, Shanghai 200237, China; 3 College of Materials and Textile Engineering, Jiaxing University, Jiaxing 314001, China; yyr0515@zjxu.edu.cn; 4 State Key Laboratory of Proteomics, Beijing Proteome Research Center, National Center for Protein Sciences (Beijing), Institute of Lifeomics, Beijing 102206, China; chenpeiru12@126.com; 5 Shanxi Key Laboratory of Biomedical Metal Materials, College of Materials Science and Engineering, Taiyuan University of Technology, Taiyuan 030024, China; hangruiqiang@tyut.edu.cn; 6 Australia-China Centre for Tissue Engineering and Regenerative Medicine, Queensland University of Technology, Brisbane, QLD 4000, Australia; yin.xiao@qut.edu.au; 7 Department of Orthopedics, Shanghai Jiaotong University School of Medicine, Shanghai 200233, China

**Keywords:** osteocyte, nanoscale, osteogenesis, TGF-β, osseointegration

## Abstract

Osteocytes function as the master orchestrator of bone remodeling activity in the telophase of osseointegration. However, most contemporary studies focus on the manipulation of osteoblast and/or osteoclast functionality via implant surface engineering, which neglects the pivotal role of osteocytes in de novo bone formation. It is confirmative that osteocyte processes extend directly to the implant surface, but whether the surface physicochemical properties can affect the functionality of osteocytes and determine the fate of the osseointegration in the final remodeling stage remains to be determined. Titania nanotube arrays (NTAs) with distinct diameters were fabricated to investigate the relationship between the nanoscale topography and the functionality of osteocytes. In vitro results pinpointed that NTA with a diameter of 15 nm (NTA-15) significantly promote osteogenesis of osteocytes via the enhancement of spreading, proliferation, and mineralization. The osteocyte transcriptome of each group further revealed that the TGF-β signaling pathway plays a pivotal role in osteocyte-mediated osteogenesis. The in vivo study definitely mirrored the aforementioned results, that NTA-15 significantly promotes bone formation around the implant. Consequently, nanoscaled topography-induced osteocyte functionality is important in late osseointegration. This suggests that surface designs targeting osteocytes may, therefore, be a potential approach to solving the aseptic loosening of the implant, and thus strengthen osseointegration.

## 1. Introduction

Late aseptic loosening during osseointegration is the most common reason for implant failure, causing approximately 75% of failures [1]. In addition to wear debris particles, implant migration and mechanical instability are important risk factors for aseptic loosening, where areas of unloading (stress shielding) and supraphysiological loading (periprosthetic interface) are associated with bone loss [2,3]. Implant failure leads to significant morbidity and accounts for substantial health care expenditures [4]. Current strategies to prevent the loosening mostly focus on the enhancement of osteoblast functionality or the inhibition of osteoclast activity [2]. Additionally, recent studies have shown that early blood clots and the initial immune response play pivotal roles in the process [5,6]. However, osseointegration is highly sophisticated in a dynamic spatiotemporal way, involving multiple cells and biological responses, which implies that the research should not only focus on the early process.

Osteocytes, the most abundant cells in bone, are terminally differentiated cells of the osteoblast lineage that are embedded in the mineralized matrix [7]. During osseointegration, osteocytes can be regarded as indicators of bone quality at the bone–implant interface [8]. Although they reside within confined spaces, denoted as lacunae, they play critical roles in bone formation and remodeling [9]. Osteocytes contribute to bone formation through activation of the SOST/Sclerostin mechanism, whereas osteocyte-manipulated bone remodeling occurs through the signaling mechanism involving the receptor activator of nuclear factor-κB ligand (RANKL), RANK, and osteoprotegerin (OPG) [10,11]. Additionally, osteocytes embedded in the mineralized bone matrix surrounding orthopedic implants can sense mechanical stimulation within the lacunar–canalicular system and convert the mechanical stimuli into anabolic or catabolic signals [12,13]. Accordingly, osteocytes orchestrate the balance between bone formation by osteoblasts and resorption by osteoclasts, and thus determine the binding capacity with the implant. However, the interaction between osteocytes and implant surface has not been well characterized, which will contribute to the etiological explanation of aseptic loosening.

Commercially pure titanium (Ti) and its alloy are among the most widely used clinical implant materials [14]. However, under in vivo conditions, Ti and its alloys are bioinert, and thus lack bioactivity to enhance the osseointegration. Accordingly, a variety of implant design features has been investigated in recent decades, ranging from macroporous geometries to surface modifications on the micron-, submicron-, and nanoscales [8,15,16,17]. Among them, surface nanoscale topography on Ti-based implants has been heavily evidenced as an effective approach to improving their biological performance [18]. Titania nanotube arrays (NTAs) decorated on Ti are attractive nanostructures, due to the advantageous features of proven biocompatibility, thermal stability, and corrosion resistance [19]. Additionally, the simplicity of achieving unique nanotube architectures with controllable dimensions at the nanoscale means that NTAs are desirable for a range of biomedical applications [5]. Advances in materiobiology indicate that cellular behaviors are inherently sensitive to local surface characteristics [20]. For instance, NTAs with a small diameter are shown to not only promote the initial vitality of endothelial cells and angiogenesis, but also activating macrophages to a pro-healing M2 phenotype via the downregulation of inflammatory-related gene expression [17]. However, whether the behavior of osteocytes can be affected by NTAs of a distinct nanoscale topography remains unknown. Accordingly, we present NTAs with distinct diameters (ranging from 15 to 120 nm), aiming to determine the impact of surface nanoscale topography on the functionality of osteocytes and the underlying mechanism.

## 2. Results and Discussion

### 2.1. Characterization of NTAs with Distinct Nanoscale Topography

**Figure 1:**
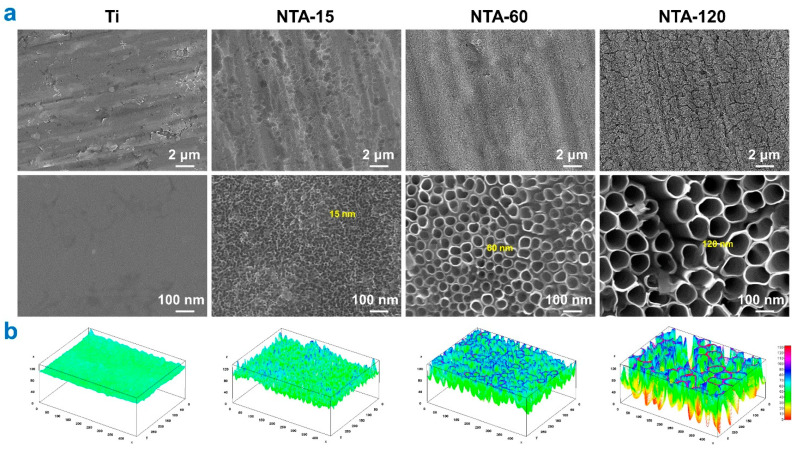
Surface topography of Ti and NTAs. (**a**) Representative SEM images of Ti and NTAs. (**b**) Surface 3D profiles of Ti and NTAs.

Figure 1a displays the surface morphology of Ti and NTAs. The surface of Ti is flat and smooth at high magnifications. Highly ordered NTAs with distinct diameters were acquired after one-step anodization. The average diameters of NTAs were around 15, 60, and 120 nm, thus denoted as NTA-15, NTA-60, and NTA-120, respectively, hereafter.

Distinct surface characteristics could be verified in the 3D profile (Figure 1b), where NTA-15 shows a larger specific surface area compared with that of NTA-120. Except for the surface morphology, the roughness and wettability are of importance to the biological responses [21,22]. In our previous study, we demonstrated that the surface roughness on Ti, especially NTA-120, was much higher than that of NTA-15 and NTA-60, whereas the wettability of the surfaces displayed a contrasting result: NTA-120 exhibited strong hydrophilicity [5]. Thus, NTA-15 showed a lower surface roughness and hydrophobicity tendency.

### 2.2. Biocompatibility and Osteogenesis Capacity of Osteocytes on NTAs

**Figure 2:**
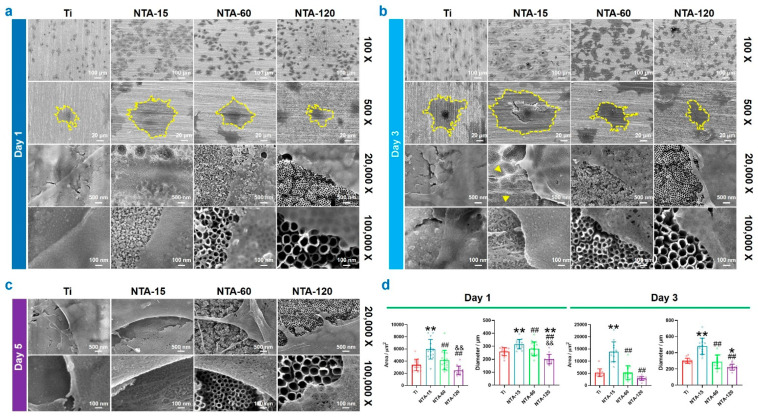
Osteocyte morphology on Ti and NTAs. (**a**–**c**) Representative images of osteocyte cultured on each group for 1, 3 and 5 days, respectively. (**d**) Qualitative results of single cell spreading area after cultured on each group for 1 and 3 days. * *p* < 0.05 compared with Ti, ** *p* < 0.01 compared with Ti, ^##^ *p* < 0.01 compared with NTA-15, ^&&^ *p* < 0.01 compared with NTA-60.

Osteocytes are long-lived, highly interconnected, terminally differentiated osteoblasts that reside within the mineralized bone matrix [23]. They constitute about 95% of adult bone cells and exert important functions, including in the regulation of bone remodeling, phosphate homeostasis, and mechanical stimuli sensing and response [12]. It has been evidenced that osteocytes directly extend to the implant and sense the surface physicochemical properties [24], i.e., bone quality around the implant is closely related to the functionality of osteocytes. However, the impact of surface nanoscale topography on osteocytes remains unconfirmed. Herein, osteocytes are incubated on Ti and NTAs to investigate whether the surface topography can induce different degrees of osteogenesis tendencies of osteocytes in vitro. Figure 2 depicts the cell adhesion and spreading condition after culturing the specimens for 1, 3, and 5 days. Low-magnification images (the upper two lines in each figure) show that all four specimens are support the adhesion and spreading of osteocytes. On day 1, osteocytes cultured on NTA-15 exhibited an extended area and flat lamellipodia compared with Ti (Figure 2a). Extension of culture time to 3 days induced abundant cell-to-cell contacts on the surface of NTA-15, and the differences in lamellipodia are more pronounced (Figure 2b). Numerous pseudopodia could be observed in NTA-15 (yellow arrow), whereas others showed a limited and flat pattern. After incubation for 5 days, the results were consistent with the above description that NTA-15 supports the spreading of osteocytes (Figure 2c). Conducting further quantitative results of single-cell area and diameter, depicted in Figure 2d, revealed that NTA-15 significantly promoted the spreading of osteocytes in the initial stage.

**Figure 3:**
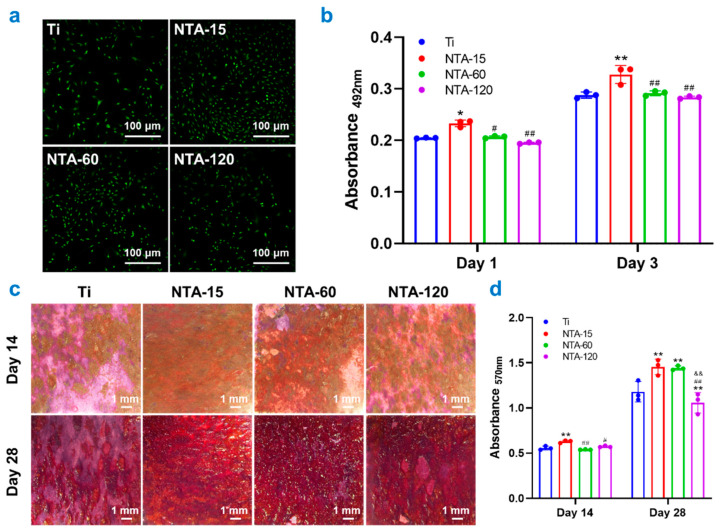
In vitro biocompatibility and osteogenesis of osteocytes on Ti and NTAs. (**a**) Live/dead staining of osteocytes cultured on each group after 1 day. (**b**) MTT results of osteocytes cultured on each group after 1 and 3 days. (**c**,**d**) Qualitative images and quantitative results of mineralization incubated on each group for 14 and 28 days. * ^#^ *p* < 0.05 compared with Ti, ** *p* < 0.01 compared with Ti, ^##^ *p* < 0.01 compared with NTA-15, ^&&^ *p* < 0.01 compared with NTA-60.

Live/dead staining was performed to assess cell viability and showed no evidence of dead (red) cells on any of the surfaces at any time point (Figure 3a). Additionally, the number of adhered osteocytes on the surface of NTA-15 was significantly higher than that of other groups. The metabolic activities of osteocytes were assessed by an MTT assay (Figure 3b), and showed that NTA-15 had a prominent effect on the proliferation of osteocytes compared with the other groups at 1 and 3 days. The qualitative and quantitative assays of ECM mineralization after osteogenic induction for 14 and 28 days are shown in Figure 3c and Figure 3d, respectively. These data are consistent with the aforementioned results and show that cells cultured on NTA-15 have a higher rate of ECM mineralization than other groups, i.e., NTA-15 stands out as having a greater ability to enhance osteogenic differentiation than any of the other groups. This finding is in agreement with our previous study: NTA-15 on the Ti surface results in superior biocompatibility and benefits the osteogenesis performance of bone-related cells [17]. It is evidenced that a nanoscale topology exerts a great influence on osteoblastic proliferation and differentiation [16]. This study indicated that the cells exhibited better proliferation and differentiation on the nanotubes compared with on flat Ti. Additionally, there was a size-dependent behavior for mastering the cell behaviors of initial adhesion, spreading, and differentiation of osteoblasts. It is reported that cells adhere more easily to the smaller diameter nanotubes instead of the larger nanotubes, due to more proteins aggregated on the smaller diameter nanotubes [25]. In addition to the surface nanoscale topography, the improved osteogenic differentiation can also be attributed to changes in the roughness-induced wettability alternation. As documented, hydrophilicity increased with the diameter of NTAs incremented due to the increased roughness, which is consistent with the present study. Albumin, fibrinogen, and FXII are the most abundant proteins in the culture medium and represent more than 50% of all serum proteins. It has been demonstrated that these proteins are more strongly adherent to a hydrophobic surface than to a hydrophilic surface [26]. Thus, NTA-15 is highly expected to attract more protein adhesion and promote cell adhesion in combination with the nanoscale effect. Briefly, these results are a clear indication that NTA-15 with a smaller nanoscale topography promotes osteocyte spreading and proliferation, as well as osteogenic differentiation, consequently improving osteogenesis which contributes to osseointegration.

### 2.3. NTA-15 Induces Osteogenesis in a TGF-β Signaling Pathway

**Figure 4:**
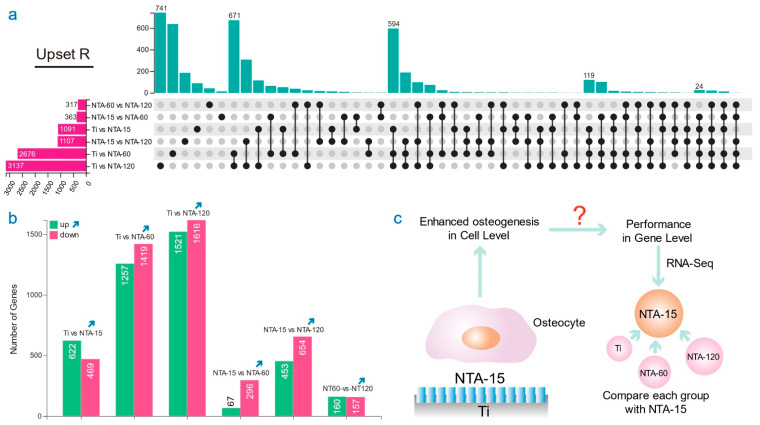
RNA-Seq results of osteocytes cultured on Ti and TNAs. (**a**) Upset R analysis of differential genes among groups. (**b**) The numbers of up-/downregulated genes among the comparison. (**c**) Illustration of the following framework of gene analysis.

The osteocyte transcriptome of the 12 total specimens was assessed to investigate the underlying mechanism of distinct nanoscale topography-induced osteogenesis. UpsetR analysis was used to assess the differential gene sets among groups [27]. It visualizes intersections of sets as a matrix, in which the rows represent the sets, and the columns represent their intersections (Figure 4a). The comparison of Ti vs. NTA-120 possesses 3137 differential genes and 741 genes are exclusive compared with other gene sets. Similarly, in the comparison of Ti with NTA-15/NTA60, both have more than 1000 differential genes, which indicates that a single alternation of surface nanoscale topography strongly impacts the gene expression of osteocytes. Figure 4b further details the upregulated and downregulated genes among groups: 622 genes were upregulated, whereas 469 genes were downregulated in the comparison of Ti with NTA-15. NTA-15 displayed a remarkable enhancement of osteogenesis; therefore, we chose this group as the targeting analysis object to determine the diversity at the gene level, and aimed to demonstrate the pivotal signaling pathway.

**Figure 5:**
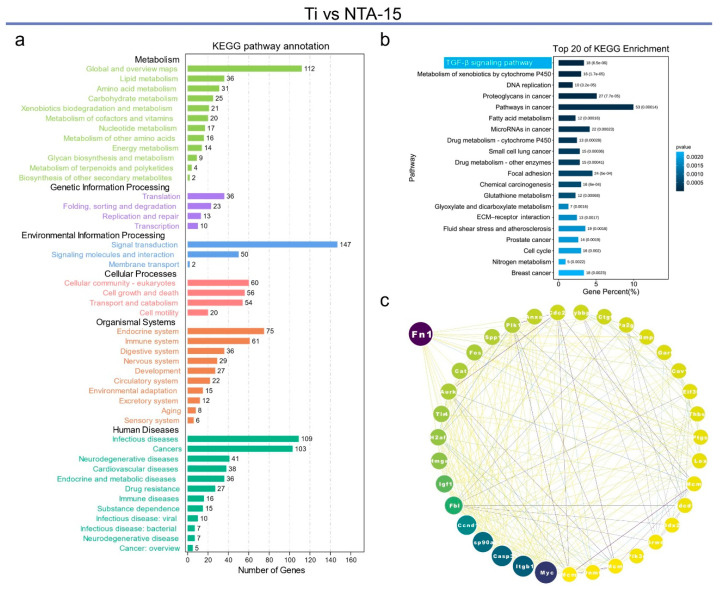
Analysis of differential genes within Ti vs. NTA-15. (**a**,**b**) KEGG pathway annotation and enrichment analysis of Ti vs. NTA-15, respectively. (**c**) Protein interaction analysis of the differentially expressed genes.

Firstly, KEGG pathway annotation was applied to the comparison of Ti with NTA-15; the result showed that most of the differential genes are enriched in signaling transduction, which is a form of environmental information processing (Figure 5a). Further data mining demonstrated that the transforming growth factor-beta (TGF-β) signaling pathway has the highest enrichment of differential genes among the top 20 KEGG annotations (Figure 5b). The TGF-β signaling pathway is important for tissue remodeling and/or repair to maintain tissue homeostasis [28]. In the bone tissue, activation of the signaling pathway can initiate bone formation and regulate bone remodeling [29]. Loss of the spatial and temporal TGF-β signaling results in several complications, including Camurati–Engelmann disease (CED) [30], Loeys–Dietz syndrome [31], Shprintzen–Goldberg syndrome [32], Marfan syndrome [33], osteogenesis imperfecta [34], and osteoarthritis [35]. Additionally, a recent study further revealed that the suppression of TGF-β signaling causes severe deterioration of the osteocyte canalicular network and dysregulates the expression of a host of perilacunar/canalicular remodeling genes [36]. Loss of osteocyte-intrinsic TGF-β signaling also reduces bone matrix mineralization. These findings further confirm that the fulfilled osteogenesis of osteocytes on NTA-15 is induced by the TGF-β signaling pathway. The genes involved in these pathways were introduced to String and Cytoscape to further investigate the interactions at the protein level (Figure 5c). The most abundant link is directed to fibronectin 1 (Fn1). Fn1 is an evolutionarily conserved glycoprotein found in all tissues of the body and functions in several stages of fracture healing [37]. Fn1 acts as a three-dimensional scaffold immediately following trauma, guiding the assembly of additional ECM components [38]. Moreover, Fn1 regulates cellular behavior via integrin-binding and growth-factor-binding domains, promoting downstream responses including cell recruitment, proliferation, and differentiation. Additionally, it is documented that Fn1 overexpression activates the TGF-β/PI3K/Akt pathway and promotes collagen production [39].

**Figure 6:**
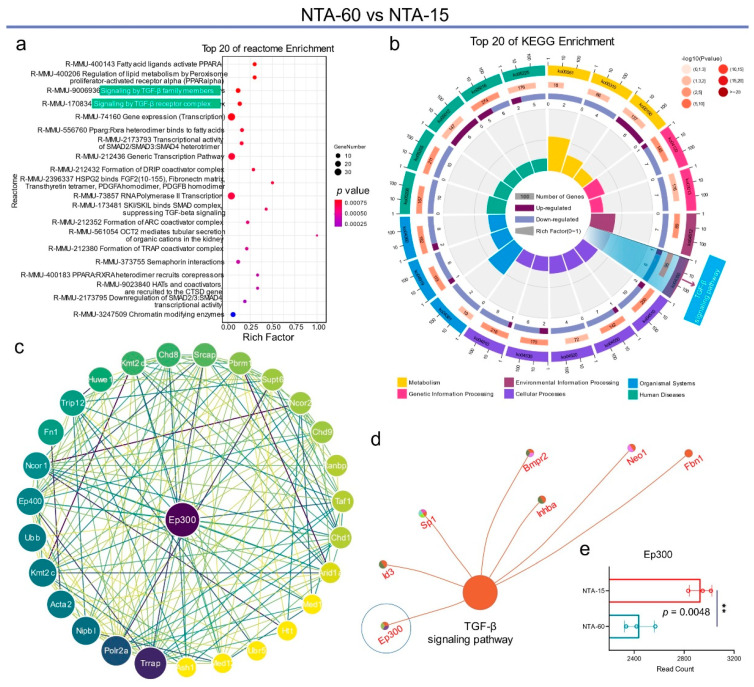
Analysis of differential genes within NTA-60 vs. NTA-15. (**a**) KEGG pathway enrichment analysis of NTA-60 vs. NTA-15. (**b**) Circular enrichment of KEGG pathways. (**c**) Protein interaction analysis of the differentially expressed genes. (**d**) Differential genes in the TGF-β signaling pathway. (**e**) The gene expression profile of Ep300. ** *p* < 0.01.

**Figure 7:**
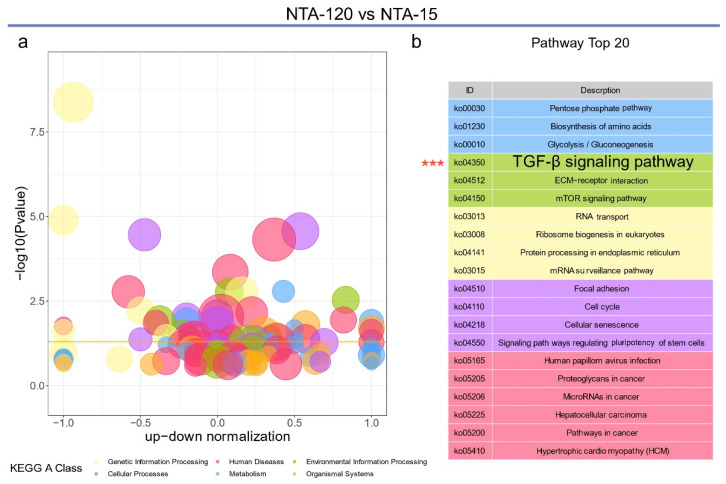
Analysis of differential genes within NTA-120 vs. NTA-15. (**a**) Bubble chart of the KEGG pathway enrichment analysis of NTA-60 vs. NTA-15. (**b**) The detailed list of Top 20 KEGG enrichment pathways. The asterisks highlight the TGF-signaling pathway.

Similar results were obtained in the comparisons of NTA-60 vs. NTA-15 and NTA-120 vs. NTA-15 (Figure 6 and Figure 7, respectively). The TGF-β signaling pathway exhibited the highest enrichment of differential genes in the top 20 Reactome (Figure 6a) and KEGG annotations (Figure 7b). Furthermore, these genes were enriched in signaling transduction, which is a form of environmental information processing (Figure 6b and Figure 7a). Notably, the most abundant link of the differential proteins was directed to E1A binding protein p300 (EP300) in the comparison of NTA-60 with NTA-15 (Figure 6c). Further data mining indicated that Ep300 belongs to the TGF-β signaling pathway (Figure 6d), and its expression in the group of NTA-15 is significantly higher (*p* = 0.0048) than that of NTA-60. Ep300 is a type of histone acetyltransferase that is essential for the activity and stability of Runx2 [40]. It participates in the transduction of signals elicited by several regulatory factors, including TGF-β and the Wnt signaling pathway [41]. In brief, NTA-15 significantly activates the TGF-β signaling pathway of osteocytes compared with other groups, which benefits the osteogenesis of osteocytes and further late stage of osseointegration.

### 2.4. In Vivo Verification of Fulfilled Osseointegration with NTA-15

**Figure 8:**
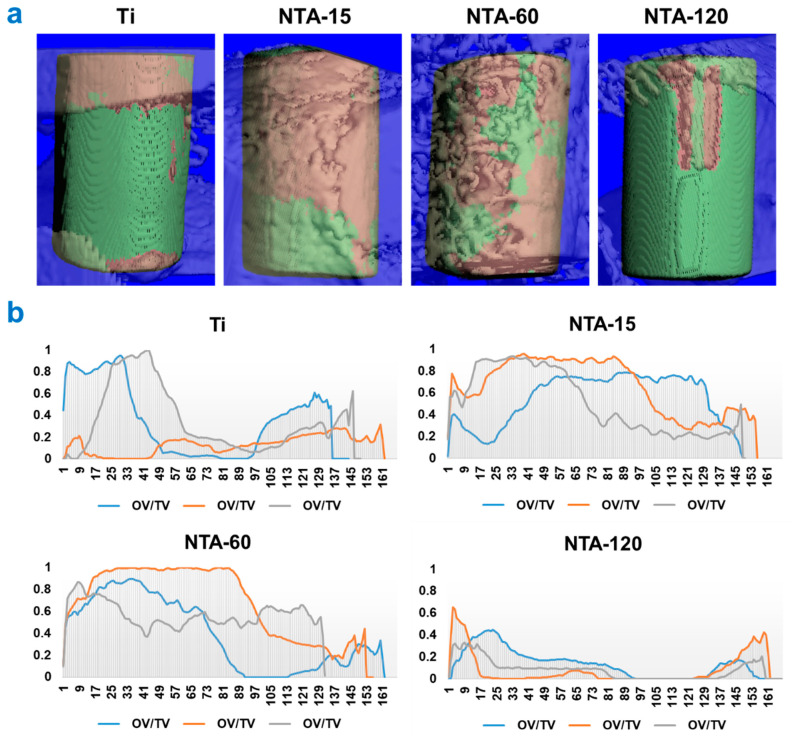
In vivo osseointegration of Ti and TNAs. (**a**) Three-dimensional rebuilt images of the implants and the peri-implant bone tissue; (**b**) original BIC profiles of each specimen obtained from the micro CT.

Animal trials were conducted for eight weeks after implant placement to verify the long-term impact of the implants on osseointegration in vivo. Three-dimensional reconstructions of the implants depicted that de novo bone formation around NTA-15 is remarkably higher (brown area) than that of other groups (Figure 8a). Notably, the volume of new bone formation gradually decreased with the enlargement of the nano-dimension. Figure 8b illustrates the volume profile per scan of the implants in vivo. The curve chart reflects the normal bone–implant contact (BIC) near the implants, which are original data of each group obtained from the μ-CT. Approximate 120 slices were scanned, and the results show that NTA-15 enables great BIC compared with that of other groups. In contrast, bone formation near the surface of NTA-120 is suppressed as the BIC decreased quickly at the initial stage. In brief, NTA-15 significantly enhances the de novo bone formation, and thus, the osseointegration, which is consistent with the in vitro results.

**Figure 9:**
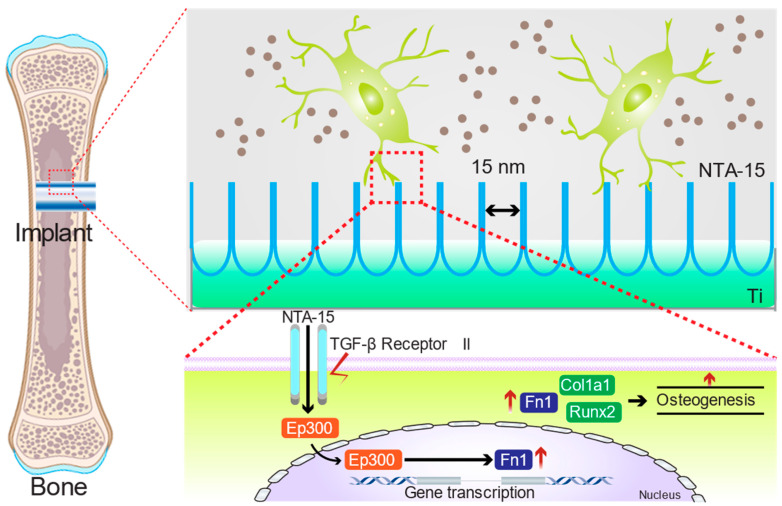
Schematic illustration of NTA-15-mediated TGF-β signaling pathway activation in the amelioration of osteogenesis.

The effect of the Ti and NTAs on the osteocyte functionality and osseointegration is illustrated in Figure 9, to elucidate the essence of the surface nanoscale topography-induced osteogenesis of osteocytes. At the late stage of osseointegration, the embedded osteocytes near the implant will sense the surface nano-dimensions and respond. NTA-15 with a smaller diameter and a higher surface specific area is capable of activating the TGF-β signaling pathway through its receptor, thus facilitating the proliferation and differentiation of osteocytes through the osteogenesis-related cytokines (e.g., Runx2, Col1a1), consequently fulfilling osseointegration.

Overall, the present study reveals potential interactions between surface nanoscale topography and osteocytes, which will develop the current understanding of de novo bone formation and osseointegration at the late stage. Most contemporary studies prevent aseptic loosening by focusing on the early stage such as immune response, osteogenesis, and angiogenesis; therefore, there is negligence of the late stage that surface-characteristic-manipulated functionality of osteocytes plays a fundamental role in osseointegration. The current study might present a distinctive approach of targeting the aseptic loosening, which may elucidate the discordances between in vitro and in vivo studies.

## 3. Materials and Methods

### 3.1. Surface Modification of Ti

Ti foils with a dimension of 10 mm × 10 mm × 0.3 mm and Ti rods with a length of 5.0 mm and diameter of 3.0 mm were used in this study. Before anodization, the specimens were ultrasonically cleaned in acetone, ethanol, and ultrapure water sequentially. Highly ordered NTAs with distinct diameters were applied to the substrates using an electrochemical cell (IT6120, ITECH) with a two-electrode configuration. Ti specimens were used as the anode electrode, whereas platinum foil was set as the counter electrode. Electrochemical treatments were carried out in ethylene glycol solution containing 0.5 wt.% ammonium fluoride (NH_4_F), 5 vol% methanol, and 5 vol% distilled water. The applied potentials were 5 V for 2 h, 30 V for 1 h, and 60 V for 10 min separately at room temperature. Subsequently, the as-prepared specimens were ultrasonically cleaned in ethanol for 10 min and air-dried.

### 3.2. Surface Characterization of the Specimens

The surface morphology of the Ti and NTAs was characterized by field-emission scanning electron microscopy (FE-SEM, JSM-7100, JEOL) at an accelerating voltage of 15 kV. The average diameter of NTAs was determined by the Digimizer. The 3D profile was determined by the plugin-surface plot in ImageJ software.

### 3.3. Cell Culture

Mouse osteocyte-like cell line-IDG-SW3 was purchased from the cell bank of the Shanghai Institutes for Biological Sciences. This cell line enabled us to study the properties of osteocytes and examine their biological and pathological function. IDG-SW3 cells were cultured on type I collagen-coated plates in α-MEM (Gibco, Life Technologies, Carlsbad, CA, USA) supplemented with 10% fetal bovine serum (Gibco, Life Technologies, Carlsbad, CA, USA) and 1% penicillin–streptomycin, in an incubator with 5% CO_2_ at 37 °C. The culture medium was changed every two days.

### 3.4. Biocompatibility Evaluation and Osteogenesis Activity of Osteocytes on the Specimens

Cell morphology after culturing on the specimens for 1, 3, and 5 days was determined by FE-SEM. Cell proliferation was evaluated using MTT after culturing for 1 and 3 days. Osteogenic differentiation, as measured by extracellular matrix (ECM) mineralization, was induced by applying an osteogenic induction medium.

### 3.5. Transcriptome Analysis of the Osteocytes Cultured on the Specimens

To demonstrate the effect of surface nanoscale topography on the functionality of osteocytes, the cells were collected after incubated on the specimens for 7 days. Total RNAs were extracted and the sequencing platform of BGI-500 (BGI, Shenzhen, China) was applied to obtain the whole gene expression profiles. Quality control checks were performed to confirm sequencing saturation and gene mapping distribution. Values of fragments per kilobase of transcript per million mapped reads (FPKM) were applied to express relative gene abundance. Normalized standard deviations, also known as the coefficients of variation, were used to quantify the sensitivity or stability of gene expression among groups. Read count data were standardized, and the significance and fold-change (*p* < 0.001 and log_2_ fold change > |1|) were set; the differences in expression were analyzed by DEGseq. The genes significantly expressed in the comparison of Ti vs. NTA-15, NTA-60 vs. NTA-15, and NTA-120 vs. NTA-15 were introduced to KEGG pathway enrichment, and a network structure was developed through the String database and Cytoscape software.

### 3.6. In Vivo Verification of NTAs in the Promotion of Osteointegration

This work was carried out following protocols approved by the Animal Research Committee of the Institutional Animal Care and Use Committee of SHCQ (SHCQ-20200017). A total of 6 male New Zealand white rabbits, aged around 10 months with a weight of ~3.0 kg, were used for the in vivo trials. The animals were premedicated through general anesthesia by intramuscular injection. Four cylindrical titanium rods were implanted in each distal surface of the bilateral femoral condyles of the animal. All surgical operations were performed under a strict aseptic protocol. At the time point of 8 weeks, the animals were euthanized, and the femurs were immediately harvested and fixed in 4% paraformaldehyde. The bone tissue containing the implant was first introduced to μ-CT (Skyscan 1176, Skyscan, Bremen, Germany) to analyze the bone mass. The regions of interest (ROIs) were selected near the growth plate. Three-dimensional reconstructions of the ROIs were finalized by CTVol software (Skyscan, Bremen, Germany).

### 3.7. Statistical Analysis

Quantitative data displayed herein were processed in triplicates and are expressed as the mean ± standard error of the mean. One-way analysis of variance (ANOVA) and Student–Newman–Keuls (SNK) post hoc tests were applied to determine the statistical significance in GraphPad Prism 8. Values of *p* < 0.05 and *p* < 0.01 were statistically significant and highly significant, respectively.

## 4. Conclusions

The fundamental role of surface nanoscale topography-induced osteocyte functionality in the late stage of osseointegration has been unraveled herein. We disclose that NTA-15 can significantly promote the spreading, proliferation, and osteogenic differentiation of osteocytes in vitro and in vivo. The process is dependent on the TGF-β signaling pathway via the transduction of Ep 300 and Fn1. The observations not only highlight the importance of nano-dimensions on osteocytes behavior during the late osseointegration, but also present a distinctive approach for targeting aseptic loosening from the viewpoint of osteocytes, aiming to elucidate the discordances between in vitro and in vivo studies.

## Data Availability

The data that support the findings of this study are available from the corresponding author.
